# Adrenal Steroids Modulate Fibroblast-Like Synoviocytes Response During *B. abortus* Infection

**DOI:** 10.3389/fendo.2019.00722

**Published:** 2019-10-22

**Authors:** María Virginia Gentilini, Guillermo Hernán Giambartolomei, María Victoria Delpino

**Affiliations:** Instituto de Inmunología, Genética y Metabolismo (INIGEM), Universidad de Buenos Aires (UBA), Consejo Nacional de Investigaciones Científicas y Técnicas (CONICET), Buenos Aires, Argentina

**Keywords:** DHEA, cortisol, *Brucella*, synovial fibroblast (FLS), RANKL

## Abstract

*Brucella abortus* stimulates an inflammatory immune response that stimulates the endocrine system, inducing the secretion of dehydroepiandrosterone (DHEA) and cortisol. In humans, the active disease is generally present as osteoarticular brucellosis. In previous studies we showed that *B. abortus* infection of synoviocytes creates a proinflammatory microenvironment. We proposed to determine the role of cortisol and DHEA on synoviocytes and infiltrating monocytes during *B. abortus* infection. Cortisol inhibited IL-6, IL-8, MCP-1, and MMP-2 secretion induced by *B. abortus* infection in synovial fibroblast. Cortisol-mediated MMP-2 inhibition during *B. abortus* infection was reversed by IL-6. DHEA inhibited *B. abortus-*induced RANKL up-regulation in synovial fibroblast through estrogen receptor (ER). *B. abortus* infection did not modulate glucocorticoid receptor (GR) expression. Cell responses to cortisol also depended on its intracellular bioavailability, according to the activity of the isoenzymes 11β-hydroxysteroid dehydrogenase (HSD) type-1 and 11β-HSD2 (which are involved in cortisone-cortisol interconversion). *B. abortus* infection did not modify 11β-HSD1 expression and GRα/β ratio in the presence or absence of adrenal steroids. Supernatants from *B. abortus*-infected monocytes induced 11β-HSD1 in synovial cells. Administration of cortisone was capable of inhibiting the secretion of RANKL by synoviocytes mimicking cortisol's effect. These results go along with previous observations that highlighted the ability of synovial tissue to secrete active steroids, making it an intracrine tissue. This is the first study that contributes to the knowledge of the consequence of adrenal steroids on synoviocytes in the context of a bacterial infection.

## Introduction

Brucellosis is an infection caused by bacteria of the genus *Brucella*. This is one of the infectious diseases transmissible between animals and humans. *Brucella* osteoarthritis is one of the most common features of human brucellosis. The most frequent joint involvements are spondylitis, arthritis, and osteomyelitis (1, 2). The acute and the chronic forms of human brucellosis present joint involvement. Clinical characteristics include joint pain, which increased local warmth, tenderness, and limitation of movement. In brucellar arthritis, cartilage loss, and bone erosion affecting different joints may eventually lead to permanent joint dysfunction (3, 4). In about 50% of the cases of osteoarciular brucellosis, bacteria are isolated from synovial fluid samples. In the affected joint, the synovial membrane may present a lymphomononuclear infiltrate in the chronic phase of the disease, but usually this takes place in the acute setting (5, 6).

Synovial damage caused by *Brucella* infection involves different immune mechanisms. We have demonstrated that *Brucella* infects and survives within human synoviocytes, and this infection elicits a proinflammatory microenvironment with the secretion of interleukin (IL)-6 and the chemokines IL-8; chemoattractant of neutrophils and monocyte chemoattractant protein 1 (MCP-1); chemoattractant of monocytes; and the secretion of matrix metalloproteases (MMPs) and RANKL—with concomitant osteoclastogenesis (7, 8).

During *Brucella* infection different cytokines generated, including those produced in the local osteoarticular site, exerted a direct effect on immune or bone cells and also influenced indirectly these cells through their capacity to influence several neuroendocrine mechanisms, including the stimulation of the hypothalamus-pituitary-adrenal axis (HPA) (9). A cross-regulation between adrenal steroids (glucocorticoids and dehydroepiandrosterone [DHEA]) and the immune response modulation (10) has been established. The effects of DHEA are frequently opposed by the adrenal steroid cortisol (11). Further, in the course of immune response, hormones are endogenously released. The type of immune response that humans develop against Brucella infection is influenced by glucocorticoids and DHEA. Accordingly, it has been demonstrated that in patients with acute brucellosis, cortisol levels were more elevated than those of healthy individuals (12, 13). In addition, we have previously demonstrated that steroid hormones are implicated in the modulation of osteoblast differentiation and macrophage response during *B. abortus* infection (13, 14). In synoviocytes, the link between inflammation and the endocrine system at local level may be due to the presence of functional receptors for glucocorticoids, androgens, and estrogens.

The potential mechanism that is involved in synoviocytes and bone damage during *Brucella* infection has been partially deciphered (7, 8). Considering our previous results which demonstrate an inappropriate secretion of steroid hormones in patients with acute brucellosis (12, 13), the aim of this work was to determine if this hormonal dysregulation is implicated in the development and evolution of osteoarticular disease.

To this end we investigated the consequence of cortisol and DHEA on synoviocyte responses during *B. abortus* infection.

## Methods

### Bacterial Culture

*Brucella abortus* S2308 was grown overnight in 10 ml of tryptic soy broth (Merck, Buenos Aires, Argentina) with constant agitation at 37°C. To prepare the bacteria inocula, we performed the procedure previously described (14). All live *Brucella* manipulations were carried out in biosafety level 3 facilities located at the at the Instituto de Investigaciones Biomédicas en Retrovirus y SIDA (INBIRS).

### Cell Culture

The immortalized human FLS cell line SW982 was obtained from the ATCC (Rockville, MD). The SW982 cell line was cultured in an α-Minimum Essential Medium (α-MEM) (Gibco) supplemented with 2 mM L-glutamine, 10% heat-inactivated fetal bovine serum (FBS) (Gibco), 100 U/ml penicillin, and 100 μg/ml streptomycin. The human monocytic cell line THP-1 was cultured in RPMI 1640 medium (Gibco) supplemented with 2 mM L-glutamine, 10% heat inactivated FBS, 100 U/ml penicillin, and 100 μg/ml. The cultures were maintained in a 5% CO2 atmosphere at 37°C.

### Cellular Infection

SW982 at a concentration of 3 × 10^5^ cells/well (for cytokine determination by ELISA) and at 5 × 10^4^ cells/well (for intracellular survival assay) were seeded in 24-well plates, and at 5.2 × 10^5^ cells/well (for mRNA extraction) it was seeded in 6-well plates. It was infected at different multiplicities of infection (MOI) in the presence or absence of DHEA (1 × 10^−8^ M) and cortisol (1 × 10^−6^ M) and incubated for 1 h at 37°C in a 5% CO_2_ atmosphere. Cells were extensively washed with DMEM-F12 to remove extracellular bacteria and were incubated in medium supplemented with 100 μg/ml of gentamicin and 50 μg/ml of streptomycin to kill extracellular bacteria in the presence or absence of DHEA and cortisol at the indicated concentrations. SW982 cells and culture supernatants were harvested at 24 h to obtain whole cell extracts and determine cytokines, chemokine production, matrix metalloproteinase (MMP) secretion, and mRNA extractions. To determine *Brucella* intracellular survival, cells were lysed with a sterile solution of 0.1% (vol/vol) Triton X-100 in H_2_O. To enumerate CFU, lysates from serial dilutions were plated on tryptic soy agar plates.

THP-1 cells were seeded at 5 × 10^5^ cells/well in 24-well plates and infected at MOI 100 for 1 h, then washed with RPMI and incubated during 24 h with medium supplemented with antibiotics as was described above.

Neutralization experiments were performed with anti-TNF receptor (anti-TNFRc, BD biosciences) at a concentration of 20 μg/ml. Synoviocytes were preincubated with the anti-TNFRc neutralizing antibody (20 mg/ml) or its corresponding isotype controls for 1 h at 37°C, and then stimulated with supernatants from *B. abortus*-infected monocytes.

Fulvestrant treatment was performed by using a concentration of 10 μM of Fulvestrant (Sigma).

### Measurement of Cytokine Concentrations

Secretion of IL-6, TNF-α, IL-1β, IL-10, IL-8, and monocyte chemotactic protein 1 (MCP-1) was quantified by enzyme-linked immunosorbent assay (ELISA; BD Biosciences, San Jose, CA) and RANKL was quantified by ELISA (R&D systems) in culture supernatants.

### Zymography

The method of Hibbs et al. with modifications was used to determine gelatinase activity (7, 15). The reversion of the inhibitory effect of cortisol on MMP production was carried out in the presence of human recombinant IL-6 at a concentration of 20 ng/ml (rhIL-6, R&D Systems).

### mRNA Preparation and Quantitative PCR

RNA was extracted using the Quick-RNA MiniPrepKit (Zymo Research) and 1 μg of RNA was subjected to reverse transcription using Improm-II Reverse Transcriptase (Promega). PCR analysis was performed with a Mx3000P real-time PCR detection system (Stratagene) using SYBR Green as fluorescent DNA binding dye. The primer sets used for amplification were: CycA sense: 5′- GCATACGGGTCCTGGCATCTTG−3′, antisense: 5′- TGCCATCCAACCACTCAGTCTTG−3′; 11β-HSD1 sense 5′- ATGATATTCACCATGTGCGCA−3′ antisense 5′- ATAGGCAGCAACCATTGGATAAG-3′; 11β-HSD2 sense 5′- TCGCGCGGTGCTCATCAC−3′antisense 5′- GTACGCAGCTCGATGGCACC−3′; GRα sense 5′- GAAGGAAACTCCAGCCAGAAC−3′ antisense 5′- GATGATTTCAGCTAACATCTCG−3′; GRβ sense 5′- GAAGGAAACTCCAGCCAGAAC−3'antisense 5′- TGAGCGCCAAGATTGTTGG−3′; DKK1 sense 5′- TCCCCTGTGATTGCAGTAAA-3′antisense 5′- TCCAAGAGATCCTTGCGTTC-3′.

The amplification cycle for GRα and GRβ was 95°C for 15 s, 65°C for 30 s and 72°C for 60 s while for 11β-HSD1 and DKK1 it was 95°C for 15 s, 62,5°C for 30 s and 72°C for 60 s. The fold change (relative expression) in gene expression was calculated using the relative quantitation method (2^−ΔΔCt^). Relative expression levels were normalized against CycA.

### Statistical Analysis

Statistical analysis was performed with one-way analysis of variance, followed by the *post-hoc* Tukey test, using GraphPad Prism 5.0 software. The data are represented as means ± standard error of the mean (SEM).

## Results

### Cortisol and DHEA Modulate *B. abortus* Intracellular Replication in Synovial Cells

**Figure 1:**
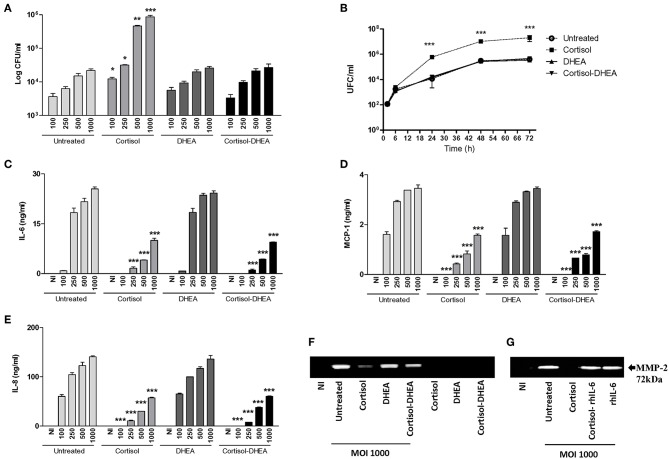
Adrenal steroids modulate *B. abortus* intracellular replication, cytokines, and chemokine secretion by synoviocytes. **(A)** After infection at different multiplicities of infection (MOI, 100 to 1000) in the presence or absence of cortisol (1 × 10^−6^ M), dehydroepiandrosterone (DHEA) (1 × 10^−8^ M), or cortisol plus DHEA (1 × 10^−6^ and 1 × 10^−8^ M, respectively); cells were incubated with antibiotics to kill extracellular bacteria. Cell lysates obtained at 24 h post-infection were plated onto agar to determine intracellular colony forming units (CFU). At 2, 6, 24, and 48 h post-infection at MOI 1000, synovial cells treated or not with cortisol, DHEA, and Cortisol-DHEA were plated on agar to determine intracellular CFU **(B)**. After 24 h post-infection IL-6, MCP-1 and IL-8 were measured in culture supernatants by ELISA **(C–E)**. MMP-2 production by *B. abortus* infected synoviocytes at MOI of 1000 was measured by gelatin zymography **(F)**. Reversion of the inhibitory effect of cortisol on MMP-2 secretion by recombinant IL-6 (rhIL-6, 20 ng/ml) **(G)**. Data are given as the mean ± SEM from at least three individual experiments. ^*^P < 0.1; ^**^P < 0.01; and ^***^P < 0.001 vs. untreated cells. NI, non-infected.

We have previously demonstrated that primary human synovial fibroblast and SW982 cell line support *B. abortus* invasion and replication (7, 8). Taking into account that adrenal steroids do not only alter the function of host cells but can also affect the intracellular replication of bacteria (13, 16, 17), we aimed to determine if these hormones could modify *B. abortus* replication in synovial fibroblast. The capacity of *B. abortus* to replicate in synovial fibroblast was significantly increased by cortisol with respect to untreated controls. In contrast, DHEA treatment had no effect. However, during the administration of cortisol and DHEA in conjunction, no differences were observed in intracellular bacterial survival with respect to untreated cells, indicating that DHEA avoided the effect of cortisol These differences were significant at 24, 48, and 72 h post-infection (Figures 1A,B). Taken together, these results indicate the intracellular replication of *Brucella* was increased by cortisol treatment whereas DHEA treatment avoided this effect.

### Cortisol Inhibits IL-6, IL-8, and MCP-1 Induced by *B. abortus* Infection in Synovial Fibroblast

*B. abortus*-infected synovial fibroblasts secrete the proinflammatory cytokine IL-6, chemokines, and MMP-2, but not the anti-inflammatory cytokine IL-10 (data not shown). In the migration of innate inflammatory cells, chemokines, and MMPs participated (18) in the concomitant tissue damage. Adrenal steroids can modulate the expression of cytokines, chemokines and MMPs in several cell types. When synovial fibroblasts were infected with *B. abortus* in the presence of cortisol, these cells secreted significantly lower quantities of IL-6, IL-8, MCP-1, and MMP-2 in respect to untreated cells. DHEA treatment could not avoid the effect of cortisol on IL-6, IL-8, and MCP-1 expression but could partially avoid the inhibitory effect of cortisol on MMP-2 expression, as was demonstrated when infection experiments were performed in the presence of both cortisol and DHEA (Figures 1B–E). Our results show that cortisol reduces the expression of secreted mediators induced by *B. abortus* infection in synoviocytes and DHEA could only partially avoid the effect in MMP-2 expression.

### IL-6 Avoids the Effect of Cortisol Inhibition of MMP-2 Secretion Induced by *B. abortus* Infection in Synovial Fibroblast

Proinflammatory cytokines have been previously implicated in MMP induction in different cell types (19–24). The role of IL-6 in the downmodulation of MMP-2 induced by cortisol in *B. abortus*-infected synovial fibroblast was determined by adding recombinant human IL-6 exogenously at the time of treatment. IL-6 was able to avoid the inhibitory effect on MMP-2 secretion induced by cortisol in *B. abortus*-infected synovial fibroblasts (Figure 1F). This indicates that cortisol downmodulated MMP-2 secretion in a mechanism that at least involved the dampening of IL-6 production.

### DHEA Inhibits *B. abortus-*Induced RANKL Up-Regulation in Synovial Fibroblast Through Estrogen Receptor (ER)

**Figure 2:**
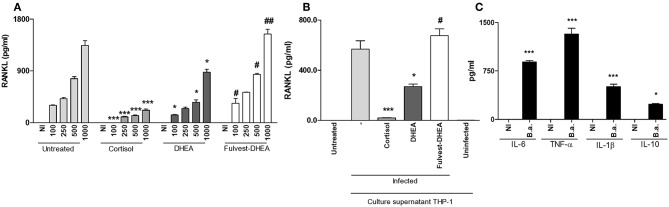
DHEA inhibits RANKL induced by *B. abortus* infection and culture supernatants from *B. abortus*-infected monocytes via estrogen receptor (ER). Synoviocytes were infected at different MOI or stimulated with 1/2 dilution of culture supernatants from *B. abortus* infected or uninfected THP-1 cells, in the presence or not of cortisol (1 × 10^−6^ M), dehydroepiandrosterone (DHEA) (1 × 10^−8^ M), and in the presence or not of the ER inhibitor, fulvestrant (Fulvest, 10 μM). RANKL was measured by ELISA in culture supernatants from *B. abortus*-infected synoviocytes **(A)** or in culture supernatants from synoviocytes stimulated with culture supernatants from *B. abortus* infected THP-1 cells (culture supernatants THP-1, infected) or culture supernatants from uninfected THP-1 cells (culture supernatants THP-1, uninfected) **(B)**. IL-6, TNF-α, IL-1β, and IL-10 levels were determined in culture supernatants from *B. abortus*-infected THP-1 cells **(C)**. Data are given as the mean ± SEM from at least three individual experiments. ^*^P < 0.1 and ^***^P < 0.001 vs. untreated cells. ^#^*P* < 0.05 and ^##^*P* < 0.01 vs. DHEA treated cells. NI, non-infected.

RANKL is the master regulator of bone metabolism involved in osteoclast differentiation (cell type implicated in bone resorption) in physiological conditions. In pathological conditions, the increase of RANKL expression could induce an exacerbation of bone resorption. We have previously demonstrated that *B. abortus* infection induced an increase of RANKL expression in synoviocytes. Then, experiments were conducted to determine if adrenal steroids could modulate RANKL expression during *B. abortus* infection in synoviocytes. To this end, infection experiments were performed in the presence of cortisol and DHEA. Cortisol treatment significantly reduced the secretion of RANKL with respect to untreated cells. In addition, DHEA treatment could also partially inhibit RANKL secretion induced by *B. abortus* infection (Figure 2A). These results indicate that adrenal hormones reduced the expression of RANKL induced by *B. abortus* infection in synoviocytes.

Infiltrating monocytes could be infected by *B. abortus* and then secrete proinflammatory cytokines and a low amount of IL-10 in response to this infection (Figure 2C) (25–27). This in turn could modulate synovial fibroblast responses (7, 27). Thus, we aimed to determine if supernatants from *B. abortus*-infected monocytes could modulate RANKL expression and if this response could be modified by the presence of adrenal steroids. Our results indicate that supernatants from *B. abortus*-infected monocytes induce RANKL expression by synoviocytes. When stimulation experiments were performed in the presence of cortisol, the expression of RANKL was completely abrogated. DHEA was able to reduce the levels of the expression of RANKL induced by supernatants from *B. abortus*-infected monocytes (Figure 2B). These results indicate that RANKL expression induced by supernatants from *B. abortus*-infected monocytes was inhibited by cortisol and DHEA treatment.

Most of the action of DHEA is mediated through ER (28). Then, experiments were conducted to evaluate the role of ER in the inhibition of the effect of *B. abortus* infection or supernatants from *B. abortus*-infected monocytes on RANKL expression in synoviocytes. To this end, fulvestran-mediated ER inhibition was employed to investigate the role of ER in regulating the DHEA effect on RANKL expression in synoviocytes infected with *B. abortus* or stimulated with conditioned medium. Fulvestrant was able to avoid the effect of DHEA in the inhibition of RANKL in *B. abortus*-infected synoviocytes or when stimulated with supernatants from *B. abortus*-infected synoviocytes (Figures 2A,B). Therefore, these results indicate that DHEA modulates the secretion of RANKL by synoviocytes mainly through ER.

## *B. abortus* Infection Induces dickkopf-1 (DKK1) Expression in Synoviocytes

**Figure 3:**
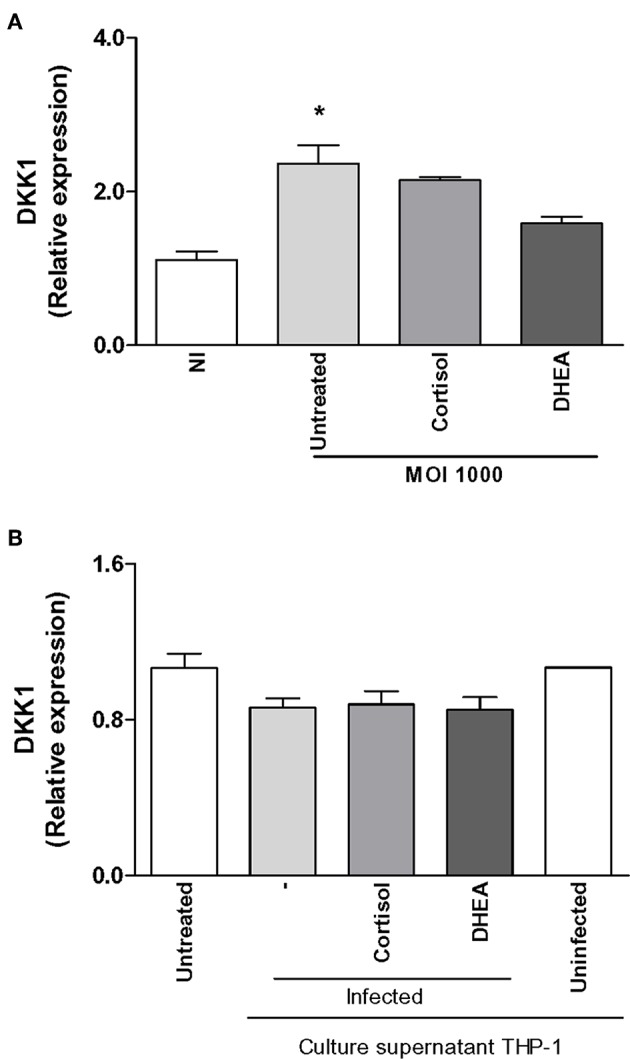
Effect of *B. abortus* infection and culture supernatants from *B. abortus*-infected monocytes on dickkopf-1 (DKK1) expression in synoviocytes. DKK1 expression was determined by RT-qPCR in synoviocytes infected by *B. abortus* at a MOI of 1000 or stimulated with 1/2 dilution of culture supernatants from *B. abortus*-infected or uninfected THP-1 cells, in the presence or not of cortisol (1 × 10^−6^ M) and dehydroepiandrosterone (DHEA) (1 × 10^−8^ M) for 24 h. Data are given as the mean ± SEM from at least three individual experiments. ^*^P < 0.1 vs. untreated infected cells. NI, non-infected.

A main factor involved in the regulation of bone biology is DKK-1, and it is deemed a contributing factor in bone resorption (29). Since DKK-1 is produced by synoviocytes, experiments were conducted to determine if *B. abortus* infection could induce DKK-1 expression and the ability of adrenal steroids to modulate this response. *B. abortus* infection induced DKK-1 expression with respect to uninfected cells (Figure 3A). Cortisol and DHEA had no effect on the expression of DKK-1 in *B. abortus*-infected cells at least at 24 h (Figure 3A). It has been previously shown that inflammatory mediators can induce the expression of DKK-1. *B. abortus*-infected macrophages secrete proinflammatory cytokines (25–27). Thus, we decided to investigate if supernatants from *B. abortus*-infected monocytes could induce DKK-1 expression by synoviocytes. Supernatants from *B. abortus*-infected monocytes failed to induce DKK-1 expression as was determined at 24 h post-stimulation. Cortisol and DHEA had no effect on this response (Figure 3B). Taken together, our results indicated that *B. abortus*-infected synoviocytes express DKK-1 that could contribute to bone damage and *B. abortus*- infected monocytes did not contribute to DKK-1 expression at the mentioned time of stimulation. In addition, in these conditions, cortisol and DHEA did not participate in the modulation of DKK-1.

### *B. abortus* Infection Does Not Modulate Cortisol Intracellular Bioavailability in Synovial Fibroblasts

**Figure 4:**
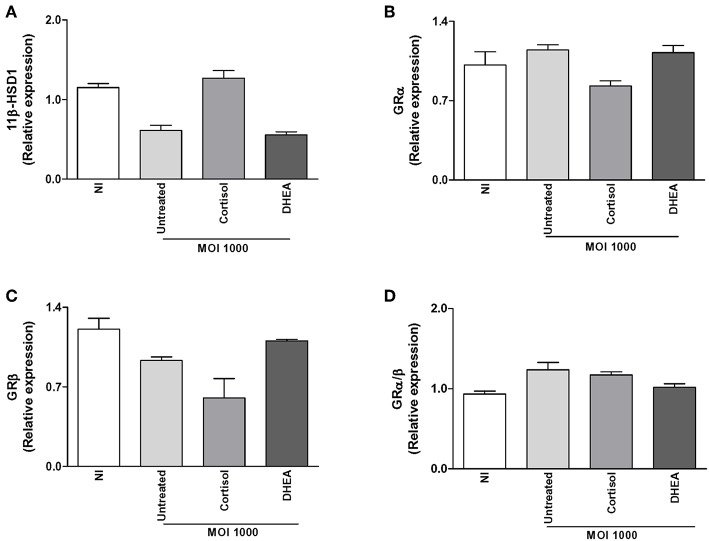
Adrenal steroids do not modulate glucocorticoid receptor (GR) and 11β-hydroxysteroid dehydrogenase (HSD)-1 during *B. abortus* infection. 11β-HSD1, GRα, and GRβ expression were determined by RT-qPCR in *B. abortus*-infected synoviocytes at MOI of 1000 in the presence or not of cortisol (1 × 10^−6^ M) and dehydroepiandrosterone (DHEA) (1 × 10^−8^ M) for 24 h. 11β-HSD1 **(A)**, GRα **(B)**, GRβ **(C)**, GRα/β ratio **(D)**. Data are given as means ± SEM from at least three individual experiments. NI, non-infected.

The capacity of cells to respond to cortisol depends not only on levels of circulating cortisol, but it is also dependent on GR expression and its intracellular bioavailability. This depends on the activity of the isoenzymes 11β-hydroxysteroid- dehydrogenase type 1 (11β-HSD1) and type 2 (11β-HSD2) that catalyze the interconversion of inactive cortisol (cortisone) to active cortisol and vice versa, respectively. Thus, experiments were conducted to establish if *B. abortus* infection could modulate the two GR receptor isoforms, termed GRα and GRβ; as well as 11β-HSD1 and 11β-HSD2 expression and to establish if this phenomenon could be modulated by adrenal steroid treatment during the infection. *B. abortus* infection did not induce the expression of GRα, GRβ, and 11β-HSD1, with respect to uninfected cells (Figure 4). In addition, adrenal steroids were unable to modulate the expression of GRα, GRβ, and 11β-HSD1 during *B. abortus* infection. In concordance of previous reports, 11β-HSD2 was not detectable in synovial fibroblast (30). These results indicated that *B. abortus* infection did not modulate GR and 11β-HSD1 in synoviocytes.

### Adrenal Steroids Modulate 11β-HSD1, GRα, and GRβ Expression in *B. abortus*-Infected Monocytes

**Figure 5:**
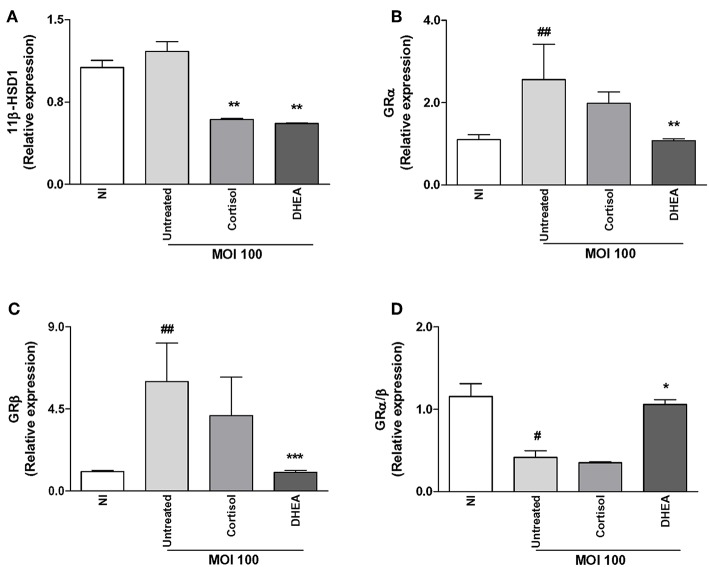
Effect of adrenal steroids on glucocorticoid receptor (GR) and 11β-hydroxysteroid dehydrogenase (HSD)-1 expression in *B. abortus*-infected monocytes. 11β-HSD1, GRα, and GRβ expression were determined by RT-qPCR in *B. abortus*-infected THP-1 cells at MOI of 100 in the presence or not of cortisol (1 × 10^−6^ M), dehydroepiandrosterone (DHEA) (1 × 10^−8^ M) for 24 h. 11β-HSD1 **(A)**, GRα **(B)**, GRβ **(C)**, GRα/β ratio **(D)**. Data are given as the mean ± SEM from at least three individual experiments. ^*^P < 0.1; ^**^P < 0.01; and ^***^P < 0.001 vs. untreated cells: ^#^*P* < 0.1; and ^##^*P* < 0.01 vs. untreated infected cells. NI, non-infected.

*Brucella*-infected synovial fibroblasts have the ability to secrete MCP-1, a key cytokine involved in monocyte migration, and monocytes could be attracted to the site of infection and contribute to modulate synovial responses. Experiments were then performed to determine if *B. abortus* infection could modulate 11β-HSD1, 11β-HSD2, GRα, and GRβ expression in THP-1 monocytes. *B. abortus* infection did not induce 11β-HSD1 expression in monocytes (Figure 5A). Infection experiments in the presence of cortisol or DHEA indicated that both steroids were able to reduce 11β-HSD1 expression. In accordance with previous results by others, the type 2 enzyme, 11β-HSD2, which converts cortisol to cortisone, was not detectable in monocytes (31).

*B. abortus* infection was also able to induce GRα and GRβ expression in monocytes. When infection experiments were performed in the presence of adrenal steroids, our results indicate that cortisol had no significant effect on GRα and GRβ expression. In contrast, DHEA was able to reduce GRα and GRβ up to basal levels (Figures 5B,C). When we analyze the GRα/β ratio, our results demonstrate that *B. abortus* infection induced a reduction of GRα/β ratio, the treatment with cortisol had no effect, and the infection experiments in the presence of DHEA increased the GRα/β ratio up to basal levels present in uninfected cells (Figure 5D).

Together, our results indicate that *B. abortus* infection did not increase the ability of the cells to respond to cortisol since it did not significantly increase 11β-HSD1 expression or the GRα/β ratio.

### Supernatants From *B. abortus*-Infected Monocytes Modulate GR and 11β-HSD1 Expression in Synoviocytes

As was mentioned before, infiltrating monocytes could be infected by *B. abortus* and secrete proinflammatory cytokines in response to this infection that could modulate synovial fibroblasts responses. Thus, we aimed to determine if supernatants from *B. abortus*-infected monocytes could modulate GRα, GRβ, and 11β-HSD1 expression in synovial fibroblasts.

**Figure 6:**
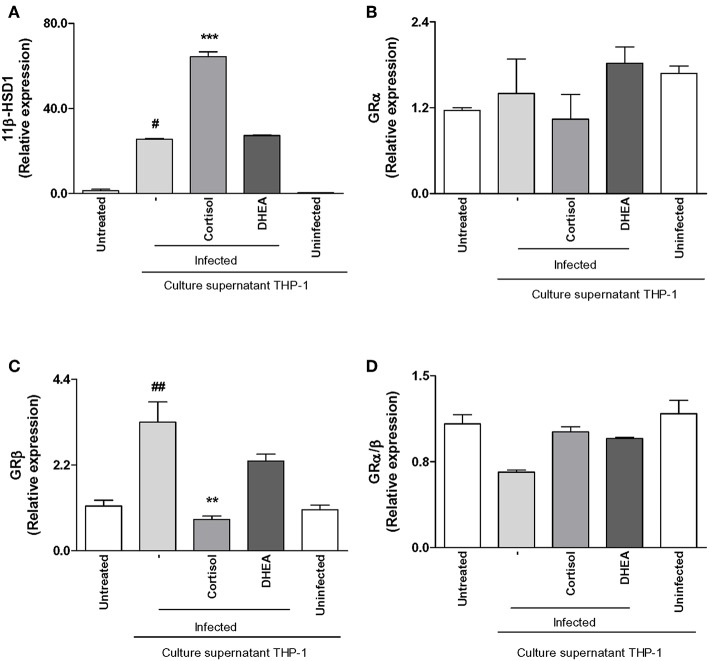
Adrenal steroids modulate 11β-hydroxysteroid dehydrogenase (HSD)-1 and glucocorticoid receptor (GR) in synoviocytes stimulated with culture supernatants from *B. abortus*-infected monocytes. 11β-HSD1, GRα, and GRβ expression were determined by RT-qPCR in synoviocytes stimulated with 1/2 dilution of culture supernatants from *B. abortus* infected or uninfected THP-1 cells, in the presence or not of cortisol (1 × 10^−6^ M) and dehydroepiandrosterone (DHEA) (1 × 10^−8^ M) for 24 h. 11β-HSD1 **(A)**, GRα **(B)**, GRβ **(C)**, GRα/β ratio **(D)**. Data are given as means ± SEM from at least three individual experiments. ^**^P < 0.01, and ^***^P < 0.001 vs. absence of cortisol. ^#^*P* < 0.1; and ^##^*P* < 0.01 vs. non-infected culture supernatant THP-1.

Supernatants from *B. abortus*-infected monocytes induced an increase of 11β-HSD1 with respect to cells stimulated with supernatants from uninfected monocytes. Cortisol significantly increased the induction in 11β-HSD1 mRNA transcription induced by supernatants from *B. abortus*-infected monocytes; in contrast, DHEA had no effect (Figure 6A). When we analyzed the modulation of supernatants from *B. abortus*-infected monocytes on the expression of GR in synoviocytes, our results indicate that supernatants from *B. abortus*-infected monocytes did not modulate GRα expression in synovial fibroblasts (Figure 6B). In addition, when stimulation with *B. abortus*-infected monocytes was performed in the presence of cortisol and DHEA, our results indicated that cortisol and DHEA had no effect. When we analyzed the expression of GRβ, supernatants from *B. abortus*-infected monocytes induced GRβ expression (Figure 6C). The presence of cortisol was able to inhibit the stimulatory effect of supernatants from *B. abortus*-infected monocytes on GRβ expression. In contrast, the presence of DHEA had no effect. It is well-known that GRβ lacks the capacity to bind glucocorticoids, and it seems to act as an inhibitor of GRα-mediated transcriptional activation through the formation of GRα/GRβ heterodimers (32). In this context, stimulation with supernatants from *B. abortus*-infected monocytes did not have a significant effect on GRα/β ratio, and the treatment with cortisol or DHEA had no effect (Figure 6D).

### Supernatants From *B. abortus*-Infected Monocytes Induce 11β-HSD1 Expression Through TNF-α

TNF-α is abundant in sites of osteoarticular inflammation (33). At the local level it has been described that TNF-α modulates 11β-HSD1 in order to convert cortisone in their active form cortisol (34).

**Figure 7:**
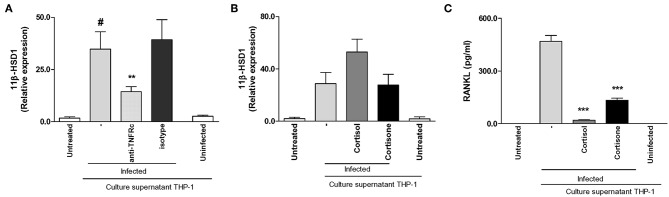
Supernatants from *B. abortus*-infected monocytes induce 11β-hydroxysteroid dehydrogenase (HSD)-1 expression in a mechanism that is dependent on the presence of TNF-α. Synoviocytes were stimulated with 1/2 dilution of culture supernatants from *B. abortus* infected or uninfected THP-1 cells in the presence or not of cortisol (1 × 10^−6^ M), or cortisone (1 × 10^−6^ M) in the presence or not of anti-TNF receptor neutralizing antibody (anti-TNFRc) at a concentration of 20 μg/ml for 24 h; and 11β-HSD1 expression was determined by RT-qPCR **(A,B)**. RANKL was determined in culture supernatants by ELISA **(C)**. Data are given as means ± SEM from at least three individual experiments. ^*^P < 0.1, vs. anti-TNFRc. ^***^P < 0.001 vs. cortisol and cortisone treatment. ^#^*P* < 0.1 vs. non-infected culture supernatant THP-1. NI, non-infected.

Then, we asked if the increment of 11β-HSD1 transcription observed in synoviocytes treated with supernatants from *B. abortus*-infected monocytes was mediated by TNF-α. To this end, stimulation of synoviocytes with supernatants from *B. abortus*-infected monocytes was performed in the presence of an anti-TNFRc-blocking antibody. TNFRc-blocking antibody significantly inhibits the expression of 11β-HSD1, whereas an isotype control had no effect (Figure 7A). These results indicated that TNF-α secreted by *B. abortus*-infected monocytes could be involved in the induction of 11β-HSD1.

### Cortisone Fails to Enhance 11β-HSD1 but Inhibits RANKL Induced by Supernatants From *B. abortus*-Infected Monocytes

We demonstrated that supernatants from *B. abortus*-infected monocytes induce 11β-HSD1 expression, and this expression is increased when stimulation was performed in the presence of cortisol. Thus, we decided to determine the effect of added cortisone instead of cortisol with culture supernatants from *B. abortus*-infected monocytes on 11β-HSD1 expression in synoviocytes. To this end, stimulation experiments were performed by added culture supernatants from *B. abortus*-infected monocytes, and after 24 h of stimulation they were treated with cortisone or cortisol as a control. After 24 h, cells were harvested to determine the expression of 11β-HSD1. Addition of cortisone to cells stimulated with culture supernatants from *B. abortus*-infected monocytes had no effect on 11β-HSD1 expression with respect to cells stimulated with supernatants from *B. abortus*-infected monocytes alone (Figure 7B). However, cortisone inhibits RANKL secretion induced by supernatants from *B. abortus-*infected monocytes (Figure 7C). These results indicate that although supernatants from *B. abortus*-infected monocytes were able to induce 11β-HSD1, cortisone was unable to enhance the expression of 11β-HSD1 but mimicked the effect of cortisol on the modulation of RANKL expression.

## Discussion

The immune system does not respond in isolation. The immune system along with the endocrine axis and the neural system act together to fight diseases. Adrenal glands secrete cortisol, a glucocorticoid hormone that plays a role in the stress response, and DHEA that has frequently opposing actions to cortisol (35).

Although the role of adrenal steroids on synovial cells has been previously studied, the impact in the context of bacterial infection has not been elucidated until now. In this context, in brucellosis, it has been previously shown that this infection elicits an imbalance in the cortisol/DHEA ratio that could impact the immune response (12, 13). Our results indicated that in synoviocytes, cortisol treatment increased *B. abortus* intracellular proliferation. This phenomenon was avoided when both cortisol and DHEA were administrated in conjunction. This increase in bacterial load has been previously described during intracellular infection of other bacteria—*Salmonella typhimurium, Mycobacterium tuberculosis*, and inclusive *B. abortus* (13, 14, 16, 17). However, the mechanisms involved in the increase of intracellular growth were not completely elucidated. While some hormones induce and increase in bacteria virulence factor expression in *Mycoplasma hyopneumoniae, Vibrio parahaemolyticus*, and *Candida albicans* (36–38), cortisol was unable to induce an increase of virulence factors in *Salmonella typhymurium* (16).

Accordingly, with its opposite effect with respect to cortisol, DHEA treatment avoided the effect of cortisol, as was revealed in cells treated with cortisol and DHEA in conjunction. On the other hand, cortisol was able to promote *B. abortus* infection not only through its intracellular replication but also by the inhibition of the immune response and cell function in synoviocytes (39). In agreement with other observations, cortisol suppressed proinflammatory mediator secretion by *B. abortus-infected* synoviocytes, and DHEA was able to partially avoid this effect at least for MMP-2. These results again support the antagonist effect of DHEA (11). The role of proinflammatory cytokines on MMP induction has been extensively reported in osteoarticular diseases (19–24). *B. abortus* infection induces IL-6 but not TNF-α and IL-1β production by synovial cells. Since rhIL-6 was able to reverse the inhibitory effect of cortisol on MMP-2 secretion in *B. abortus*-infected synovial cells, it suggests that IL-6 could be involved in such induction.

In osteoarthritis, osteoclast formation is enhanced by proinflammatory cytokines from infiltrating immune cells but also synoviocytes enhance osteoclast formation via expression of RANKL (40). During osteoarticular brucellosis, the expression of RANKL could be increased by *B. abortus* infection and by the proinflammatory environment created by *B. abortus*-infected monocytes. In this context, DHEA was able to inhibit RANKL expression. The role of DHEA in the modulation of RANKL expression was described in the context of inflammatory non-infectious osteoarticular disease (41, 42). In addition, DHEA mediated this effect through ER receptor as evidenced when infections and the treatment with supernatants from *B. abortus*-infected monocytes were performed in the presence of fulvestrant. The ability of DHEA and its metabolites to signal through androgen receptor (AR) and ER has been previously described (43, 44). However, since the addition of ER antagonist, fulvestrant, completely avoids the effect of DHEA on RANKL expression, we could affirm that the main signaling of DHEA during *B. abortus* infection in sinoviocytes is via ER.

The presence of functional ER in synoviocytes might link the endocrine system and inflammation at the local level (45). The ability of DHEA to regulate RANKL through ER has been demonstrated previously at least in osteoblast cells (44). This indicated that ER could play a role in the modulation of osteoclastogénesis through the modulation of the key molecule implicated in osteoclastogenesis, RANKL in *B. abortus*-infected synoviocytes.

DKK-1 is the master regulator of bone remodeling in osteoarticular inflammatory disease (46). DKK-1 expression inhibits osteoblast differentiation and increases osteoclastogenesis with concomitant bone resorption. *B. abortus* induces the increase of DKK-1 expression in synoviocytes; this is in concordance with the bone resorption observed in patients with osteoarticular brucellosis. However, supernatants from *B. abortus*-infected monocytes were unable to induce DKK-1 expression. This could be explained, at least in part, by the role of TNF-α, IL-6, and IL-1β present in culture supernatants from *B. abortus-*infected monocytes on DKK-1 expression. While TNF-α suppresses bone formation by inducing DKK-1, IL-6, and IL-1β have been reported as inhibitors of DKK-1 expression (47, 48). Then, the cytokines combined in the culture supernatants from *B. abortus*-infected monocytes were unable to induce DKK-1 expression.

The effect of cortisol in synovial cells does not dependent on an increase of their bioavailability, as was demonstrated by the non-modification of 11β-HSD1 and GRα/β ratio in response to *B. abortus* infection in the presence or absence of adrenal steroids. A similar situation was found for *B. abortus*-infected monocytes in which *B. abortus* infection had no effect on 11β-HSD1 but cortisol and DHEA treatment inhibited its expression in the context of the infection. In addition, *B. abortus* infection in the presence or not of cortisol inhibited GRα/β ratio and DHEA could avoid this effect. In synoviocytes and monocytes, the absence of detectable expression of 11β-HSD2 deserves to be discussed since this enzyme is involved in the conversion of cortisol in its inactive form, cortisone. The absence of negligible levels of 11β-HSD2 in human monocytes, macrophages and dendritic cells has been reported (31, 49). In synovial cells, a high expression of 11β-HSD1 has been demonstrated, mainly in synovial fibroblast, whereas 11β-HSD2 is primarily restricted to synovial macrophages (30). Taking into account that we evaluated the effect of *B. abortus* infection in synovial fibroblast, our findings are in line with previous results.

**Figure 8:**
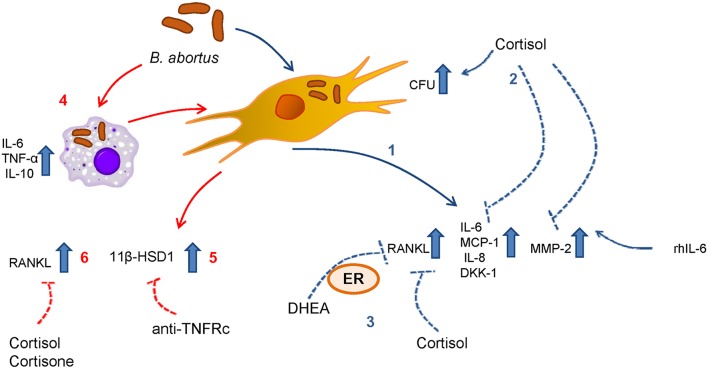
Scheme summarized the results for the mechanisms involved in the modulation of synoviocytes by adrenal steroids during *B. abortus* infection. (1) Infection with *B. abortus* induces the secretion of RANKL, IL-6, MCP-1, IL-8, DKK-1, and MMP-2. (2) When cortisol is present, the intracellular CFU is increased with respect to untreated cells. (3) DHEA and cortisol avoid the increase of RANKL induced by *B. abortus* infection. The action of DHEA is mediated through the ER. (4) *B. abortus* infection induces the secretion of IL-6, TNF-α, and IL-10 by monocytes. (5) Supernatants from *B. abortus*-infected monocytes induce 11β-HSD1 in a mechanism that is dependent on TNFRc. (6) In addition, supernatants from *B. abortus*-infected monocytes induce RANKL, cortisol, and cortisone avoid this effect.

Although the ability of monocytes to induce proinflammatory cytokines in response to *B. abortus* infection has been widely demonstrated (25, 27), supernatants from *B. abortus*-infected monocytes appear to have some anti-inflammatory effect, as was revealed by the induction of 11β-HSD1 in synovial cells. Despite this, cortisone does not seem to increase the expression of 11β-HSD1 as it does cortisol. However, the administration of cortisone is capable of inhibiting the secretion of RANKL by synoviocytes mimicking the cortisol effect. This indicates the importance of the increase of 11β-HSD1 in the utilization of cortisone. This is in accordance with previous observations that highlight the ability of synovial tissue to make active steroids, and this tissue has been considered an intracrine tissue (45). The increase of 11β-HSD1 expression induced by cortisol in synoviocytes treated with culture supernatants from *B. abortus*-infected monocytes also deserves to be discussed. The interactions between proinflammatory cytokines and glucocorticoids are often antagonistic. However, in some cases it can be additive or synergistic (50–52); even this synergistic effect of glucocorticoids on the production of 11β-HSD1 in the presence of proinflammatory cytokines was previously described in synovial fibroblasts (53). Our experiments in synovial cells treated with culture supernatants from *B. abortus*-infected monocytes in the presence of a neutralizing antibody anti-TNFRc significantly reduced 11β-HSD1; however, the remaining expression of 11β-HSD1 indicates that the contribution of other proinflammatory cytokines cannot be ruled out (Figure 8).

Finally, this is the first study that contributes to the knowledge of the effect of adrenal steroids on synoviocytes in the context of a bacterial infection. Our findings reveal that DHEA could modulate some synoviocytes function. Considering that and taking into account the modulation exerted by DHEA on other cell types in bone damage (13, 14), we can conclude that antibiotic therapy with supplementation with DHEA or its derivates could be a potential new treatment in order to reduce the bone damage during osteoarticular brucellosis.

## Data Availability Statement

The datasets generated for this study are available on request to the corresponding author.

## Author Contributions

All authors were involved in the design of the study, the preparation of the manuscript, and approve the final version for publication. MG: conceptualization, methodology, validation, formal analysis, investigation, funding acquisition, writing review and editing. GG: conceptualization, funding acquisition, writing review and editing. MD: conceptualization, methodology, validation, formal analysis, investigation, funding acquisition—supervision, validation, visualization, and writing original draft.

### Conflict of Interest

The authors declare that the research was conducted in the absence of any commercial or financial relationships that could be construed as a potential conflict of interest.
